# Percutaneous Deployment of the Sinus‐SuperFlex‐DS Stent for Hybrid Stage I Palliation in Neonates Weighing ≤ 2.5 kg: A Multicenter Study

**DOI:** 10.1002/ccd.70687

**Published:** 2026-06-14

**Authors:** Johanna Hummel, Jochen Grohmann, Katarzyna Gendera, Götz Müller, Harald Bertram, Matthias Sigler, Dietmar Schranz, Christian Jux, Markus Khalil

**Affiliations:** ^1^ Department of Congenital Heart Diseases/Pediatric Cardiology Ruhr University Bochum, Heart and Diabetescenter NRW Bad Oeynhausen Germany; ^2^ Department of Congenital Heart Defects and Pediatric Cardiology University Heart Center Freiburg‐Bad Krozingen Freiburg Germany; ^3^ Department of Congenital Heart Defects and Paediatric Cardiology, TUM University Hospital German Heart Center Technical University of Munich Munich Germany; ^4^ Department of Pediatric Cardiology, Children's Heart Clinic, University Heart & Vascular Center Hamburg University Medical Center Hamburg‐Eppendorf Hamburg Germany; ^5^ Department of Pediatric Cardiology and Critical Care Medizinische Hochschule Hannover (MHH) Hannover Germany; ^6^ Department of Pediatric Cardiology University of Münster Münster Germany; ^7^ Department of Pediatric Cardiology Goethe University Clinic Frankfurt Frankfurt Germany; ^8^ Pediatric Cardiology Pediatric Heart Center Justus‐Liebig University Giessen Giessen Germany; ^9^ Department of Pediatric Cardiology University Hospital Cologne Cologne Germany

**Keywords:** duct stenting, hybrid palliation, interstage mortality, multicenter study, neonatal intervention, self‐expanding stent

## Abstract

**Background:**

Hybrid stage I palliation (HS1P) has developed as an alternative to the Norwood stage I palliation for neonates with hypoplastic left heart and related left‐sided obstructive lesions. HS1P is currently used in various clinical settings, such as single ventricle palliation, bridge to decision, bridge to biventricular repair, or destination therapy. The procedure comprises bilateral pulmonary artery banding and duct stenting, thus avoiding the need for cardiopulmonary bypass surgery, which may be particularly beneficial in high‐risk patients.

**Aims:**

The aim of this multicenter retrospective observational study was to evaluate the feasibility, procedural safety, and clinical effectiveness of percutaneous deployment of the self‐expanding sinus‐SuperFlex‐DS (SSF‐DS) stent for duct stenting as part of HS1P in neonates weighing ≤ 2.5 kg.

**Methods:**

All neonates weighing ≤ 2.5 kg who underwent percutaneous SSF‐DS implantation as part of HS1P at five German centers between August 2011 and 2019 were included. Procedural characteristics, complications, reinterventions, and outcomes were analyzed until stent explantation or death.

**Results:**

Thirty‐two patients with a median age of 11 days (range 2−37 days) and a median weight of 2200 g (1600–2500 g) at stent implantation were included. Of those, 50% were preterm and 25% had known chromosomal abnormalities. Percutaneous stent implantation was successful in all patients. It was performed exclusively via femoral access, with retrograde arterial access used in 62.5% of procedures, including infants with a minimal weight of 1900 g. No procedure‐related deaths occurred. In 25% of patients, two SSF‐DS stents were implanted during the index procedure to achieve complete duct coverage. Twelve stent‐related reinterventions were performed in 10 patients (31%), with a median time to first reintervention of 42 days (18–135 days). Implantation of more than one duct stent as part of the index procedure was associated with a higher reintervention rate (63% vs. 29%). The mean time to the subsequent surgical step did not differ between patients with and without stent‐related reintervention. The overall reintervention rate until stent removal was 63% (20/32 patients). Twenty‐five patients (78%) were successfully bridged to surgery (72% univentricular surgery, 28% biventricular repair). Median time to stent removal was 127 days (25–500 days), with a median weight of 4300 g (2800–7000 g) and a median weight gain of 2200 g (500–5400 g). In one patient, HS1P was the destination therapy. Six patients (19%) died before the subsequent surgical step. Mortality was related to severe comorbidities and the high‐risk nature of the cohort rather than the stenting procedure itself. Procedure‐related complications included atrial arrhythmias (6%) and stent migration/embolization (6%), all managed successfully without conversion to open‐heart surgery. Occlusion of the primary access vessel occurred in two patients (6%; one femoral artery, one femoral vein).

**Conclusions:**

Percutaneous deployment of the SSF‐DS stent as part of HS1P in neonates weighing ≤ 2.5 kg is feasible and associated with acceptable procedural and access‐related complication rates. This strategy successfully bridges most patients to the next planned surgical stage despite the high‐risk characteristics of this population. Close clinical monitoring during the interstage period is warranted because of the substantial rate of reinterventions, including treatment of stent stenosis and restrictive atrial communications.

## 1 Introduction

The Hybrid Stage I Palliation I (HS1P) or hybrid approach was introduced to treat newborns with hypoplastic left heart syndrome (HLHS) without the need for cardiopulmonary bypass. The procedure includes bilateral pulmonary artery banding (PAB), stenting of the arterial duct, and, if necessary, enlargement of the interatrial communication. First described by Gibbs et al. [1], unfavorable results led the Leeds group to no longer recommend this hybrid approach [2]. However, due to the more favorable outcomes in Giessen and Columbus [3], the surgical‐interventional version of the procedure has developed as an alternative to neonatal Norwood surgery [4, 5].

The HS1P is used differently across centers in terms of technique and indications [6]. While some centers consider it the treatment of choice for neonates with obstructive left heart lesions, others reserve it for patients with additional risk factors for surgical treatment, such as low body weight [6]. In the early years of HS1P, balloon‐expandable stents were routinely used for arterial duct stenting due to the lack of alternatives [7, 8]. These stents have a high radial force, allowing severely narrowed and undersized ducts to be expanded to larger diameters, but they may conform poorly to the ducts' curvature [9]. Furthermore, when these stents are delivered percutaneously, stiff long sheaths increase hemodynamic instability when crossing both right‐sided heart valves during transvenous procedures, and they require a 5 F (French) access, which limits implantation via the femoral artery.

These limitations contributed to the development of alternative approaches, including transpulmonary duct stenting, as pioneered by the Columbus group [5, 8, 10].

A major advancement in percutaneous duct stenting for duct‐dependent systemic perfusion was the introduction of the sinus‐Repo stent, a closed cell, self‐expanding stent system (9). Subsequently, the self‐expanding sinus‐SuperFlex‐DS (SSF‐DS, Optimed, Karlsruhe, Germany) was introduced, loaded into a highly flexible delivery system that can be easily advanced through a 4 F introducer sheath. This open‐cell stent device became available in 2011 and received the CE (Conformité Européenne) mark for duct stenting in newborns with HLHS.

Although self‐expanding duct stents have been used successfully for hybrid palliation and their performance has been reported previously, data specifically addressing their percutaneous deployment in neonates weighing ≤ 2.5 kg remain limited.

In this high‐risk patient population, procedural safety and successful bridging to the next stage of treatment are of particular importance. While percutaneous duct stenting avoids direct surgical access to the duct, it requires femoral vascular access and a separate catheter‐based procedure, both of which remain areas of debate in very‐low‐weight patients.

Therefore, the aim of this multicenter study was to evaluate the feasibility, procedural safety, and clinical effectiveness of percutaneous deployment of the SSF‐DS stent in low‐weight neonates undergoing HS1P.

## 2 Methods

### 2.1 Study Design and Setting

This is a multicenter retrospective observational study evaluating the feasibility, safety, and clinical outcomes of percutaneous SSF‐DS stent implantation in neonates with duct‐dependent systemic circulation and low birth weight. Eleven institutions in German‐speaking countries with interventional pediatric cardiology units were contacted to participate. Five centers contributed data (University Heart Center Freiburg, Pediatric Heart Center Giessen, University Medical Center Hamburg‐Eppendorf, Hannover Medical School, TUM University Hospital German Heart Center). The remaining centers had not treated patients who met the inclusion criteria at the start of the study.

All neonates and infants weighing 2.5 kg or less who received a percutaneously implanted SSF‐DS stent in the arterial duct from August 2011 (CE approval) until August 2019 were included in the analysis. All implantation and follow‐up data were collected as part of routine care. Frequency and modality of the follow‐up were determined individually by each participating institution.

Written informed consent was obtained for catheterization and stent implantation. Approval for retrospective data collection and analysis was granted by the local ethics committee.

Pre‐procedural data included demographic information and indication for stenting. The fluoroscopy plane used for measurements of the ducts' diameter and length, diameter of the aorta, and stent diameter was not standardized and was at the discretion of each center. During the study period, SSF‐DS stents were available in diameters ranging from 6 to 9 mm and lengths from 12 to 20 mm. The range has since been expanded to include diameters of 4 to 9 mm and lengths of 12 to 24 mm. All stent sizes are pre‐loaded into a delivery system compatible with a 4 F sheath and designed to accommodate a 0.018” guidewire.

Follow‐up data included the most recent echocardiographic and angiographic surveillance data, as well as all percutaneous procedures until the explantation of the duct stent. Clinical and procedure‐related complications were recorded. Procedural complications were classified as major if they required additional interventions or measures, and as minor if they were self‐limiting. In cases of deceased patients, the last available examination was considered the final follow‐up.

### 2.2 Outcomes

#### 2.2.1 Feasibility and Safety

The primary objective of this study was to evaluate the feasibility and safety of percutaneous deployment of the SSF‐DS stent in neonates weighing ≤ 2.5 kg.

Primary outcome measures included procedural success, procedure‐related complications, freedom from reinterventions or unplanned surgery, and survival to the next planned stage of treatment.

Stent stenosis could result from ductal tissue ingrowth, thrombosis, insufficient radial force of the stent, or somatic growth of the patient. To assess possible stent compression during follow‐up, the rate of clinically significant stent stenosis requiring intervention (percutaneous or surgical) was evaluated. Stent compression was assumed if the measured stent diameter on angiography was smaller than at the time of implantation.

The success of the palliation strategy was evaluated based on the duration of stent palliation. A potential advantage of the HS1P is the postponement of major heart surgery on cardiopulmonary bypass beyond the vulnerable neonatal period. This may increase the patients' suitability for complex surgery.

Thus, the palliation strategy was considered successful if patients survived the neonatal period (for this study defined as at least 28 days after the estimated date of birth) without the need for surgical reintervention.

#### 2.2.2 Device‐Related Outcome Measures

While the primary objective of this study was to evaluate the feasibility and safety of percutaneous SSF‐DS deployment, data regarding the behavior of duct stents in very‐low‐weight neonates undergoing HS1P are scarce. Therefore, secondary device‐related outcome measures were assessed, including immediate stent expansion, and hemodynamic effectiveness.

Immediate device performance was assessed by whether the stent achieved its nominal diameter, indicating a ratio of 1:1 between the diameter of the stented duct (sDA) and the stent's nominal diameter. The hemodynamic effectiveness of the stent palliation was evaluated using the ratio of the smallest diameter of the sDA to the diameter of the descending aorta (DAO). The sDA/DAO ratio was used to determine whether systemic circulation could be adequately supplied by the implanted stent. Ideally, the sDA should reach at least the DAO diameter (sDA/DAO ≥ 1.0).

### 2.3 Statistics

Data are presented as absolute numbers and percentages, as medians with ranges, or as means ± standard deviations, as appropriate. Statistical analyses were performed using SPSS (version 27, IBM Corporation, Armonk, NY, USA).

### 2.4 Histopathology

The tissue specimens containing the stent were fixed in formalin following surgical removal. After embedding hard resin in methylmethacrylate (Technovit 9100, KULZER & Co, Wehrheim, Germany) and hardening of the tissue bloc, slices of 0.5 mm were sectioned using a diamond cutter (300 CP, Exakt GmbH, Norderstedt, Germany). These slices were ground down to 10−30 μm using a rotational grinder (400 CS, Exakt GmbH, Norderstedt, Germany). Standard staining was performed with Richardson blue [11, 12].

## 3 Results

### 3.1 Patient Characteristics

The study included 32 patients with an equal gender ratio (Table 1). The median age and body weight at implantation of the SSF‐DS was 11 days (range 2–37 days) and 2200 g (1600–2500 g), respectively. Nineteen percent of patients were < 2000 g. Half of the cohort was preterm with a range of 30−36 weeks of gestation. The main underlying heart defect was HLHS in 40.6%, hypoplastic left heart complex (HLHC) in 21.9%, and other cardiac defects with hypoplastic or interrupted aortic arch in 37.5%. Chromosomal abnormalities were documented in 8 out of the 32 patients. All patients underwent bilateral PAB before duct stenting. Prior to the procedure, 60% of patients were on mechanical ventilation and patients were treated with prostaglandins in dosages between 2.3 and 28 ng/kg/min. One out of five centers stopped prostaglandins 4 h before the percutaneous procedure. All but one of the ducts were categorized as morphologically tubular. The minimum native duct diameter was median 4.9 mm (2–8.6 mm), the maximum diameter was 7.0 mm (4.9–9.5 mm), with a length of 14.8 mm (7−29 mm).

**Table 1:** Patient characteristics (HLHS: hypoplastic left heart syndrome).

Patient characteristics at intervention	*n* = 32
Sex, female/male, %	50/50
Age, median (range), days	11 (2−37)
Weight, median (range), kg	2.2 (1.6−2.5)
Prematurity < 37 weeks, *n* (%)	16 (50)
Diagnosis	
Typical HLHS, *n* (%)	13 (41)
HLHS variants, *n* (%)	8 (25)
Other cardiac defects with hypoplastic/interrupted aortic arch, *n* (%)	11 (34)
Chromosomal abnormality, *n* (%)	8 (25)

### 3.2 Feasibility and Safety

#### 3.2.1 Procedural Success

Procedural success was achieved in all patients. Procedures were performed via femoral access. Retrograde femoral arterial access was used in 20 patients (62.5%), including patients weighing as little as 1.9 kg. Median fluoroscopy time was 7.5 min (2–33 min) for retrograde and 12 min (4–36 min) for antegrade approaches. Stent sizes ranged from 6.0/15 to 9.0/20 mm, with 7.0/15 mm being most common. In 25% of patients, a second stent was implanted during the index procedure to ensure complete duct coverage. The duct lengths in patients with two stents varied between 13 and 29 mm.

Additional procedures during the intervention included enlargement of the atrial communication (Rashkind procedure or balloon dilation of a restrictive interatrial communication) in eight patients, valvuloplasty of a stenotic aortic valve in two patients, balloon dilation of the duct after stenting in one patient, stent implantation of an aortic coarctation in one patient, and dilation of a stenotic pulmonary vein confluence in one patient. There were no procedure‐related deaths.

#### 3.2.2 Procedure‐Related Complications

Complications during the initial procedure occurred in 12.5% of patients. These included atrial arrhythmia (*n* = 2) and stent embolization (*n* = 2).

The first patient experienced atrial arrhythmia during an attempt to dilate a stenotic pulmonary vein confluence. The other patient with atrial arrhythmia underwent balloon atrioseptostomy as an additional procedure due to a restrictive foramen ovale. In both patients the SSF‐DS stent was placed via the femoral artery and in both, the arrhythmia was successfully resolved by electrical conversion.

Stent embolization occurred in two other patients: in one, the implanted stent (7.0/15 mm, minimum native duct diameter 4.4 mm, maximum native duct diameter 7.3 mm) migrated toward the pulmonary end of the duct (antegrade access via the femoral vein) but was corrected and stabilized by implantation of a second SSF‐DS stent (8.0/20 mm). In the other patient, the stent (8.0/18 mm, minimum native duct diameter 4.4 mm, maximum native duct diameter 6 mm) embolized into the pulmonary artery (retrograde access via the femoral artery) and was successfully snared and repositioned. In both cases, embolization occurred during stent deployment. In retrospect, the stents were not adequately positioned in the aorta. Due to the small diameter of the DAO, sufficient fixation of the stents at the aortic end could not be achieved, resulting in stent displacement toward the pulmonary artery during deployment.

During follow‐up, cardiac decompensation due to significant stenosis of the duct stent was reported in two patients. The first patient was successfully treated with balloon angioplasty of the SSF‐DS stent and stenting of the atrial septum 32 days after the initial procedure. This patient was then operated 134 days after the initial stent palliation undergoing a comprehensive stage II procedure with a body weight of 5000 g. The second patient underwent balloon angioplasty of the SSF‐DS stent and balloon dilation of an aortic coarctation 44 days after the initial procedure. The planned next surgery was moved forward and performed 99 days after the initial stent palliation (biventricular repair with a body weight of 4300 g). One patient showed signs of retrograde aortic arch obstruction. Thirty‐one days after the initial stent palliation, a Norwood stage I operation was performed with a 3.5 mm modified Blalock−Taussig shunt, with the patient weighing 2800 g. The weight gain during stent palliation for this patient was 500 g.

During the follow‐up period no stent fracture, migration or embolization was reported. However, occlusion of the primary access vessel occurred in two patients (6%; one femoral artery, one femoral vein). No stent‐related complications were reported during the explantation of the stents.

### 3.3 Interstage Outcomes

#### 3.3.1 Reinterventions and Unplanned Surgery

**Figure 1:**
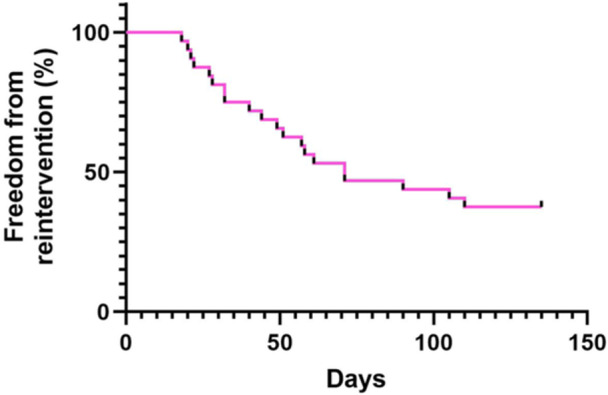
Freedom from reintervention over time. [Color figure can be viewed at wileyonlinelibrary.com]

**Figure 2:**
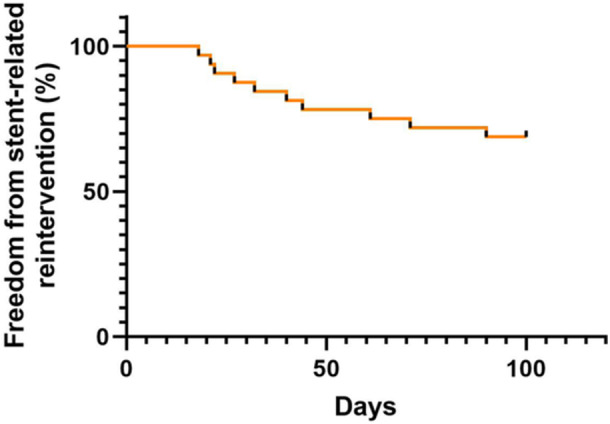
Freedom from stent‐related reintervention over time. [Color figure can be viewed at wileyonlinelibrary.com]

**Figure 3:**
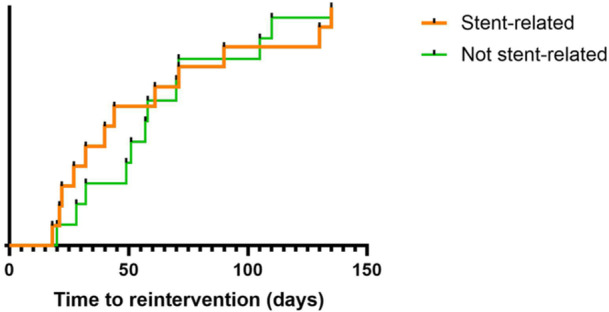
Time to first reintervention (orange: stent‐related and not stent‐related intervention, green: not stent‐related reintervention). [Color figure can be viewed at wileyonlinelibrary.com]

The median follow‐up was 121 days (12−500 days) with a complete follow‐up for all patients (100%). In 17 patients, stent stenosis of was documented either angiographically or via echocardiography. Twelve stent‐related reinterventions were performed in 10 patients, corresponding to a stenosis rate of 31%. The median time to the first reintervention was 42 days (18–135 days; Figures 1, 2, 3). The median weight at reintervention was 3000 g. The implantation of more than one duct stent during the initial procedure was associated with a higher rate for stent stenosis (29% reintervention rate in patients with a single duct stent vs. 63% in patients with two duct stents). Reinterventions included balloon angioplasty in nine patients and implantation of a second duct stent in three patients. Two patients required restenting due to incomplete duct coverage with subsequent restenosis, and one patient due to neointimal proliferation with ineffective balloon dilation. Balloon‐expandable stents were used in all patients (6/12 mm, 6/16 mm, and 7/16 mm Formula 414 stents, Cook Medical, USA) without periprocedural complications. Restenting was performed 18, 21, and 135 days after the initial procedure.

During follow‐up, reinterventions unrelated to the duct stent were performed in eleven patients. These included atrial septostomies (*n* = 5), balloon dilation (*n* = 1) or stenting of restrictive atrial communications (*n* = 4), balloon angioplasties of the pulmonary artery bands (*n* = 4), a balloon angioplasty of a branch pulmonary artery due to thrombosis, a balloon valvuloplasty of the aortic valve, and a balloon dilation of an aortic coarctation. At least one reintervention (stent‐related or not stent‐related) was required in 20 out of the 32 patients, resulting in an overall reintervention rate of 63% (Figure 1).

Unplanned surgical procedures were reported in four patients. In two of those, the Comprehensive Stage II operation was moved forward due to duct stent stenosis with cardiac decompensation after redilatation of the stented duct had already been performed. In one patient rebanding of the PAB was necessary 29 days after duct stent placement. In another patient, Norwood I surgery was performed 56 days after the HS1P due to severe stenosis of the right pulmonary artery following dislocation of the band.

#### 3.3.2 Success of the Palliation Strategy

The palliation strategy, as defined, was successful in 88% of the cohort (28 out of 32 patients), with patients surviving the neonatal period without the need for reoperation.

**Figure 4:**
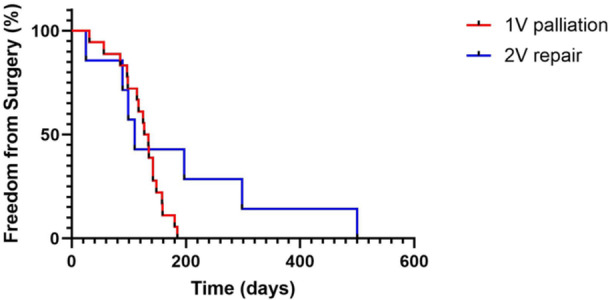
Freedom from next surgical step (red: patients with univentricular physiology (1V) treated with surgical palliation after duct stent explantation; blue: patients with biventricular physiology (2V) treated with surgical repair after duct stent explantation). [Color figure can be viewed at wileyonlinelibrary.com]

**Figure 5:**
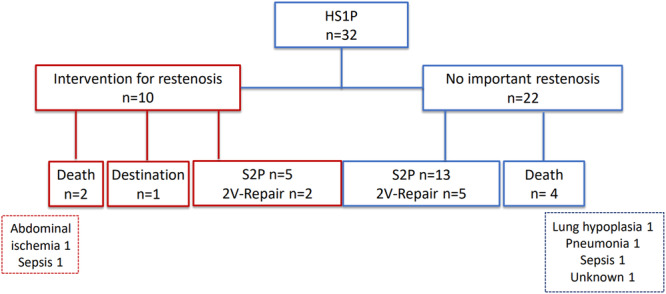
Outcome (HS1P: hybrid stage I palliation; S2P: stage II palliation; 2V: biventricular). [Color figure can be viewed at wileyonlinelibrary.com]

Overall, stent palliation resulted in surgery for 25 patients (78%), with 72% of them undergoing palliative surgery and 28% receiving biventricular repair. One patient did not have further surgery planned (Figures 4 and 5). The median time to reoperation was 127 days (25−500 days), and the median weight was 4300 g (2800−7000 g). During stent palliation, the median weight gain was 2200 g.

Among the 10 patients who experienced stent stenosis, seven had their stent palliation ended with surgery. The median time to surgery in this subgroup was 134 days (99−298 days), the median weight at that time was 4400 g (4100–7000 g). Of these, five patients underwent Comprehensive Stage II, and two patients had biventricular repair. For one patient, HS1P palliation was the destination therapy, and unfortunately, two patients died.

Among the 22 patients without significant stent stenosis, 18 underwent a subsequent surgical procedure. The median time to reoperation was 122 days (25−500 days), and the median weight was 4200 g (2800−6900 g), which did not differ from patients with stent stenosis. In this group, eight patients had a Comprehensive Stage II procedure, five underwent Norwood Stage I, and five had biventricular repair (Figure 5). Four patients in this group died.

#### 3.3.3 Mortality

There were no procedure‐related deaths. However, six patients (19%) died before the intended next surgical step could be performed. Of those, two patients had previously undergone stent‐related reinterventions. One patient with interrupted aortic arch (IAA) type B and an omphalocele received initial duct stenting at an age of 14 days, weighing 2400 g. Eighteen days later, the patient underwent reintervention because of significant stent stenosis, which was treated with balloon dilation and implantation of a second (balloon‐expandable) stent. Unfortunately, the patient died the following day due to abdominal ischemia and liver failure. It remained unclear to what extent the omphalocele contributed to the deterioration, or whether stent redilation was performed too late. Another patient, born preterm at 34 weeks gestation and small for gestational age (SGA), with bronchopulmonary dysplasia (BPD) and IAA, had initial duct stenting at the age of 37 days, weighing 2000 g. Twenty‐seven days after the procedure, the patient underwent reintervention for significant stent stenosis, treated with balloon dilation. The patient died 62 days after the second intervention due to sepsis. The other four patients did not experience significant stent stenosis. Of those, only one died within the first 28 days after the initial procedure. This patient had hypoplasia of the left lung and suffered from hypoxia alongside the congenital heart defect. He died 1 day after duct stenting at the age of 12 days due to respiratory failure. Sepsis was the cause of death in another patient with HLHS, M. Hirschsprung, and necrotizing enterocolitis (NEC), who died 58 days after stent implantation (initial stent implantation at the age of 17 days, weighing 2400 g). Pneumonia caused death in another patient with HLHS, restrictive atrial communication, SGA, who died 60 days after stent implantation and atrioseptostomy on Day 6, with a body weight of 2100 g. Finally, one patient with IAA type B, VACTERL association, and microdeletion 22q11, died 120 days after stent implantation. The procedure had been performed on Day 11, with a weight of 1950 g. The cause of death in this patient remained unknown.

### 3.4 Device‐Related Outcomes

Stenting increased the minimum duct diameter from a median of 4.6 to 5.9 mm. The sDA/stent ratio was 0.8 (0.65–1.04), meaning the stent reached 80% of its nominal diameter. According to our definition, stent palliation was hemodynamically effective in 84% of the cohort (27/32) with a sDA/DAO‐ratio of ≥ 1.0.

During follow‐up, 81% of stents were reassessed angiographically. The median time to the first reassessment by angiography was 47 days (18–130 days). As described above stenosis of the stent was documented in 17 patients either angiographically or via echocardiography. The site of stenosis was central in five, at the pulmonary end of the stent in four, and at the aortic end in three patients. Age and weight at intervention, minimum diameter of the native duct, ratio of minimum to maximum diameter of the native duct and sDA/DAO ratio did not differ significantly between the groups with and without stent stenosis (Table 2).

**Table 2:** Comparison of patients with and without stent stenosis.

	Stent stenosis (*n* = 10)	No stenosis (*n* = 22)	*p*
Age at intervention, mean (SD), days	13.7 (10.4)	11.1 (6.6)	0.40
Weight at intervention, mean (SD), grams	2170 (356)	2191 (235)	0.85
Min diameter native DA, mean (SD), mm	5.12 (1.48)	4.75 (1.26)	0.47
Ratio min/max diameter native DA, mean (SD)	0.72 (0.19)	0.71 (0.17)	0.85
sDA/DAO‐ratio	1.03 (0.23)	1.11 (0.16)	0.28

Abbreviations: DAO, descending aorta; SD, standard deviation; sDA, stented ductus arteriosus.

### 3.5 Histopathology

**Figure 6:**
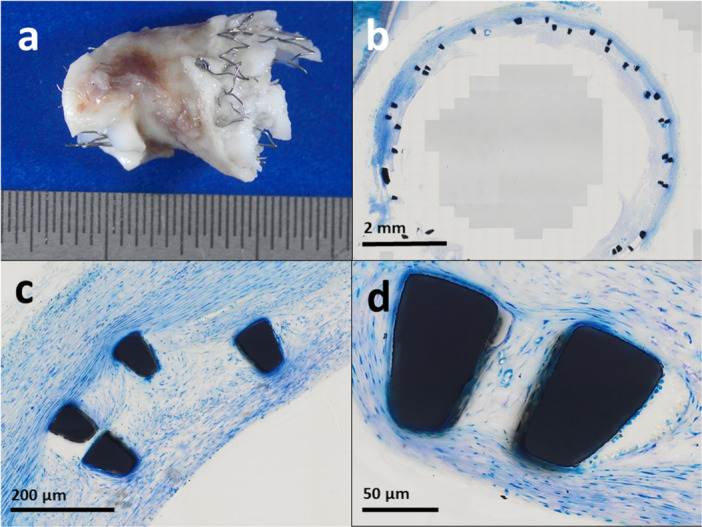
Macroscopic view of an Optimed SSF DS stent after surgical removal (a) after an implantation time of 127 days with mild to moderate pseudointima proliferation (b, c) with superficial endothelialisation but no evidence of cellular inflammatory reaction (d). [Color figure can be viewed at wileyonlinelibrary.com]

Four stent specimens were sent for histology and showed mild to moderate pseudo‐intima proliferation containing spindle shaped fibro‐muscular cells surrounded by extracellular matrix with superficial endothelialisation. No relevant cellular inflammatory reaction was detected (Figure 6).

## 4 Discussion

The principal finding of this multicenter study is that percutaneous deployment of the SSF‐DS stent as part of HS1P is feasible in neonates weighing ≤ 2.5 kg and can be performed with an acceptable procedural risk. Successful implantation was achieved in all patients, no procedure‐related deaths occurred, and 78% of patients were successfully bridged to the next surgical step. Our data provide multicenter experience with a percutaneous implantation strategy in a particularly vulnerable population of neonates. The present cohort demonstrates that this approach can be applied successfully even in infants weighing less than 2 kg. A key consideration when interpreting our findings is the alternative strategy of direct transpulmonary duct stenting via the main pulmonary artery (MPA), thereby avoiding an additional catheter‐based procedure. This approach avoids femoral vascular access and combines both components of hybrid palliation into a single intervention. In contrast, the percutaneous strategy evaluated in our study can be performed without direct surgical access to the duct and may therefore represent an alternative treatment strategy in centers with established interventional pediatric cardiology expertise.

Despite their low weight and high‐risk non‐cardiac profile, we observed no procedural deaths. Although the percutaneous strategy requires an additional catheter compared to transpulmonary duct stenting, this did not appear to have negatively impacted treatment success in our cohort. Survival and freedom from reintervention during the neonatal period were 88%.

However, six patients (19%) died before subsequent surgery, such as comprehensive stage II or biventricular repair. Five of the six deaths were attributable to severe comorbidities or non‐cardiac complications rather than the hybrid procedure itself. The Giessen group reported a combined in‐hospital and interstage mortality of 6.7% over 15 years [13]. The higher mortality rate in our cohort may reflect the high‐risk patient selection: all of the patients were low weight neonates (inclusion criteria), 28% of the patients were premature, and 25% had their cardiac defect in the context of a genetic disorder.

The overall reintervention rate was 63% including both stent‐ and not stent‐related procedures. This is also due to a high number of not stent‐related procedures, which were indicated on follow‐up, including atrial septostomy, balloon dilation or stenting of restrictive atrial communications, and balloon angioplasty of the PAB. This underscores the importance of close monitoring of the patients during follow‐up. The stent‐related reintervention rate was 31%, occurring between 18 and 135 days post‐procedure.

Although device performance was not the primary focus of this study, several observations regarding stent behavior in very‐low‐weight neonates merit discussion. One advantage of the SSF‐DS stent is its high flexibility and better alignment to the duct's shape [14]. This comes at the price of a lower radial strength, reflected in a stented DA/stent ratio of 0.8. Therefore, the use of balloon‐expandable stent should be considered for initial palliation in the setting of narrowed or severely obstructed ducts. In our study, the duct had a sufficiently large diameter after stent implantation in 84% of the 32 patients with a sDA/DAO‐ratio of ≥ 1.0. A crucial criterion for a good hemodynamic outcome is the absence of a peak‐to‐peak gradient between pulmonary artery and DAO. It is important to note that the diameter of the chosen stent should always exceed the DAO diameter by 1−2 mm [7]. This intentional oversizing strategy is intended to prevent stent migration toward the DAO, and also takes into account the limited radial strength of the stent, as well as the child's weight gain during the interstage period.

In our cohort, two cases of stent embolization into the pulmonary artery occurred where the duct stents were not sufficiently anchored on the aortic side. These events underscore the importance of adequate, yet carefully calibrated aortic stent placement, particularly in patients with small diameter of the DAO, to ensure stable positioning while preventing excessive extension into the aorta.

Goreczny et al. reported similar reintervention rates and observed differences between self‐expanding and balloon‐expandable stents, supporting the importance of ongoing surveillance after hybrid palliation [15].

Covering the whole length of the duct is crucial to avoid recurrent obstruction after termination of prostaglandin therapy [16]. Despite the wide range of available SSF‐DS stent lengths (nowadays up to 24 mm), in 25% of our cohort two overlapping stents were implanted. This is less than described by Goreczny et al. (67.8%) [15]. One important finding is, that the group with two implanted stents had more stent‐related reinterventions compared to patients with a single duct stent in this study (63% vs. 29%). This is consistent with other studies [15, 17] and should be acknowledged during interstage by close monitoring for in‐stent stenosis. In fact, follow‐up examinations on a weekly or 2‐weekly basis should be considered [18]. Patients who needed two stents in our study had a duct length between 13 and 29 mm. Besides the fact that a second stent may be required due to residual stenosis or recoil, with increasing experience the ratio of patients who need two stents to cover the whole length of the duct might drop.

A possible strategy to decrease the rate of stenosis of the stented duct is based on the hypothesis that continuation of heparin combined with prostaglandin infusion after stent implantation in a low dosage (e.g., 5−10 ng/kg/min) for a further 24−48 h may prevent an immediate duct reaction to the foreign body [19]. The role of anticoagulant and antiplatelet drugs in the postprocedural course of the hybrid procedure remains unclear. Some centers routinely use thromboprophylaxis while other centers advocate antithrombotic medication only if more than one stent is implanted [20, 21].

A particular concern regarding the percutaneous approach is the requirement for femoral vascular access in very‐low‐weight neonates. In our cohort, retrograde femoral arterial access was successfully used in 62.5% of procedures, including patients weighing less than 2 kg. Only one femoral arterial occlusion was documented. As vascular ultrasound surveillance was not performed systematically across all participating institutions, asymptomatic vascular complications may have been underrecognized. Given the high rate of reinterventions and the vulnerability of this patient population, ultrasound‐guided puncture should be strongly considered, as it reduces number of attempts, time to access, and risk of vascular complications [22].

### 4.1 Limitations

This is a multicenter observational study with the typical limitations of a retrospective report. Since patients with low body weight with HLHS, HLHC and variants are rare, the sample size remains small, as is the number of events, limiting the statistical power. The aim of the study was to evaluate the feasibility, procedural safety and clinical effectiveness of percutaneous deployment of the self‐expanding SSF‐DS stent for duct stenting in an important subgroup of newborns and infants. Since the study has no control group, we cannot draw comparisons with alternative devices, such as the self‐expanding Protégé GPS stent (Medtronic, USA), or, alternative treatment methods, such as HS1P with transpulmonary implantation of the duct stent via median sternotomy or the completely percutaneous approach with transcatheter placement of pulmonary flow restrictors.

Additional limitations deserve consideration. Due to the retrospective multicenter design, several procedural variables were not recorded systematically across all participating institutions. These include the use of ultrasound‐guided vascular access, routine ultrasound surveillance of femoral vessels following catheterization, postprocedural antithrombotic and antiplatelet treatment strategies, and the specific indications for implantation of a second duct stent during the index procedure. Consequently, the study cannot determine the impact of these factors on vascular complications, stent stenosis, or reintervention rates.

Additionally, differences in pre‐ and postprocedural approaches and varying levels of experience across participating centers may influence results. Nevertheless, the findings provide valuable real‐world insights into percutaneous duct stenting in systemic duct dependent perfusion beyond single‐center reports.

## 5 Conclusion

Percutaneous deployment of the SSF‐DS stent as part of HS1P in neonates weighing ≤ 2.5 kg is feasible and associated with acceptable procedural and access‐related complication rates. The strategy successfully bridges most patients to the next planned surgical step despite the high‐risk characteristics of this population. Close clinical monitoring is warranted following duct stenting to enable early treatment of stent stenosis and restrictive atrial communications.

## Conflicts of Interest

The authors declare no conflicts of interest.

## Data Availability

The data that support the findings of this study are available from the corresponding author upon reasonable request.
